# Fin whale *MDH-1* and *MPI* allozyme variation is not reflected in the corresponding DNA sequences

**DOI:** 10.1002/ece3.1046

**Published:** 2014-04-16

**Authors:** Morten Tange Olsen, Christophe Pampoulie, Anna K Daníelsdóttir, Emmelie Lidh, Martine Bérubé, Gísli A Víkingsson, Per J Palsbøll

**Affiliations:** 1 Evolutionary Genetics Group, Department of Genetics, Microbiology, and Toxicology, Stockholm UniversitySvante Arrhenius Väg 20C, S-106 91 Stockholm, Sweden; 2 Marine Research InstituteSkúlagata 4, IS-101 Reykjavík, Iceland; 3 MatísVínlandsleið 12, IS-113 Reykjavík, Iceland; 4 Marine Evolution and Conservation, Centre for Ecological and Evolutionary Studies, University of GroningenPO Box 11103, 9700 CC, Groningen, The Netherlands

**Keywords:** Adaptation, marine mammals, metabolic enzymes, outlier loci, population structure, selection

## Abstract

The appeal of genetic inference methods to assess population genetic structure and guide management efforts is grounded in the correlation between the genetic similarity and gene flow among populations. Effects of such gene flow are typically genomewide; however, some loci may appear as outliers, displaying above or below average genetic divergence relative to the genomewide level. Above average population, genetic divergence may be due to divergent selection as a result of local adaptation. Consequently, substantial efforts have been directed toward such outlying loci in order to identify traits subject to local adaptation. Here, we report the results of an investigation into the molecular basis of the substantial degree of genetic divergence previously reported at allozyme loci among North Atlantic fin whale (*Balaenoptera physalus*) populations. We sequenced the exons encoding for the two most divergent allozyme loci (*MDH-1* and *MPI*) and failed to detect any nonsynonymous substitutions. Following extensive error checking and analysis of additional bioinformatic and morphological data, we hypothesize that the observed allozyme polymorphisms may reflect phenotypic plasticity at the cellular level, perhaps as a response to nutritional stress. While such plasticity is intriguing in itself, and of fundamental evolutionary interest, our key finding is that the observed allozyme variation does not appear to be a result of genetic drift, migration, or selection on the *MDH-1* and *MPI* exons themselves, stressing the importance of interpreting allozyme data with caution. As for North Atlantic fin whale population structure, our findings support the low levels of differentiation found in previous analyses of DNA nucleotide loci.

## Introduction

Population genetic data have been utilized to infer intraspecific population genetic structure in ecology and conservation since the early 1960s when the advent of experimental methods enabled detection of individual genetic variation (Sick 1961). The appeal of genetic inference methods to assess population genetic structure is grounded in the correlation between the genetic similarity and gene flow among populations. This specific aspect has been utilized extensively to guide the management of natural populations where a significant level of population genetic divergence serves as the basis for delineating a species into conservation and management units (Moritz 1994; Waples and Gaggiotti 2006; Palsbøll et al. 2007). The effects of migration are typically genomewide; however, at occasion, some loci may appear as outliers displaying a substantially higher or lower degree of genetic divergence relative to the genomewide level of genetic divergence. Such signatures are usually inferred as loci subject to either divergent or balancing selection, respectively (Tajima 1989; McDonald and Kreitman 1991; Fu and Li 1993; Kreitman 2000). Divergent selection might be due to unique local adaptations (Protas et al. 2006, 2011; Storz et al. 2007, 2009; McCracken et al. 2009a; Scott et al. 2011; Nielsen et al. 2012), which in turn may warrant additional protective measures (Nielsen et al. 2009b; Allendorf et al. 2010; Ouborg et al. 2010; Hoffmann and Sgro 2011).

Allozymes are different variants of enzymes coded by the same locus (Hunter and Markert 1957; Ingram 1957; Markert and Moller 1959; Crick et al. 1961). Such expressed genetic variation is more likely to be subject to local selection and consequently detected as outliers in comparisons with selectively neutral DNA sequences, such as the mitochondrial control region or single tandem repeat (STR) loci (Ford 2002; Storz and Nachman 2003; Canino et al. 2005; Skarstein et al. 2007; Nielsen et al. 2009b). Allozyme analysis was the primary method to collect population genetic data (Hubby and Lewontin 1966; Lewontin and Hubby 1966) but was largely replaced when dideoxy-terminator nucleotide sequencing (Sanger et al. 1977), STR genotyping (Tautz 1989; Schlotterer et al. 1991) and other methods for detecting changes in the DNA sequence itself became more efficient. Recently, the increased focus on the genetics of adaptive variation in natural populations have renewed the interest in allozyme loci, as these may serve as a good starting point for detecting genomic regions under selection (Wheat et al. 2006; Hemmer-Hansen et al. 2007; Ellegren and Sheldon 2008; Nielsen et al. 2009a; Crease et al. 2011; Kirk and Freeland 2011; Schoville et al. 2012). Most studies of this kind make the implicit assumption that outlying allozyme loci are adaptive, and the different alleles arise due to nonsynonymous nucleotide substitutions in the DNA sequence coding the allozymes. However, cis-regulatory processes, such as alternative splicing of messenger RNA and/or post-translational modifications, may yield similar allozyme variation (King and Wilson 1975; Mann and Jensen 2003; Matlin et al. 2005; Marden 2008; Chen and Manley 2009; Choudhary et al. 2009; Keren et al. 2010; Kelemen et al. 2013). Assessing the relative contribution of protein-coding and cis-regulatory processes in shaping allozyme variation is not only fundamental in understanding locus evolution (Hoekstra and Coyne 2007; Carroll 2008; Barrett and Hoekstra 2011), but also central to the interpretation of allozyme variation in terms of estimating rates of gene flow and population divergence time (e.g., to delineate management units), as the observed variation may be transient and thus not represent the action of migration and/or local adaptation.

Here, we present the results of an assessment of two outlying allozyme loci detected among samples collected from North Atlantic fin whales, *Balaenoptera physalus*. The North Atlantic fin whale has been the target of multiple population genetic analyses of data collected from allozyme loci (Daníelsdóttir et al. 1991, 1992), as well as STR genotypes and mitochondrial control region sequences (Bérubé et al. 1998). Early work, based upon allozyme variation, revealed very high levels of genetic divergence among the summer feeding areas of Eastern Canada, around Iceland, Norway, and Atlantic Spain, indicative of low migration rates and substantial population structuring across the North Atlantic (Daníelsdóttir et al. 1991, 1992). In contrast, subsequent analyses of presumed selectively neutral genetic markers (the mitochondrial control region and STR loci) exhibited low levels of genetic differentiation across the North Atlantic (Bérubé et al. 1998). While intriguing in itself, this discrepancy have resulted in an unclear understanding of North Atlantic fin whale migration patterns and significantly hampered management efforts (IWC 2007, 2009).

The purpose of our study was to examine whether the variation observed in outlying allozyme loci was a result of mutations in the enzyme-encoding nucleotide sequences and thus possibly due to local adaptation. Specifically, we considered the following three possible scenarios that would result in the high levels of genetic divergence reported at the outlying allozyme loci: (1) nucleotide substitutions, possible due to divergent natural selection; (2) technical artifacts relating to differential treatment of samples during collection, storage, and processing; or (3) alternative splicing and/or post-translational modifications of the allozyme loci. To assess the possible effects of these different processes, we first re-analyzed the previously published allozyme dataset to identify the most extreme outlier allozyme loci relative to a novel dataset of 15 STR loci. Subsequently, we extracted DNA from a subset of the fin whales used in the previous allozyme study and sequenced the genes encoding the outlier allozyme loci to identify potential nucleotide substitutions that could account for the observed allozyme phenotypes (electromorphs). Surprisingly, we failed to detect any nonsynonymous substitutions in the exons encoding the outlier allozyme loci (*MDH-1* and *MPI*), suggesting that factors other than genetic drift, migration, and selection may account for electrophoretic variation in allozyme loci.

While a great number of studies in nonmodel species have contrasted population genetic divergence estimated from selectively neutral STR and mitochondrial loci with those obtained from allozyme analyses (e.g., Lemaire et al. 2000; De Innocentiis et al. 2001; Dufresne et al. 2002; Dhuyvetter et al. 2004; Vandewoestijne and Van Dyck 2010; Strand et al. 2012), few have proceeded to assess the variation at the DNA sequences encoding the divergent allozyme loci to assess the underlying molecular mechanisms (Eanes 1999; Pogson 2001; Brunelli et al. 2008; McCracken et al. 2009b; Schoville et al. 2012), and, to the best of our knowledge, none have found that the observed allozyme variation was not reflected in the corresponding DNA sequences.

## Material and Methods

### Samples

**Figure 1:**
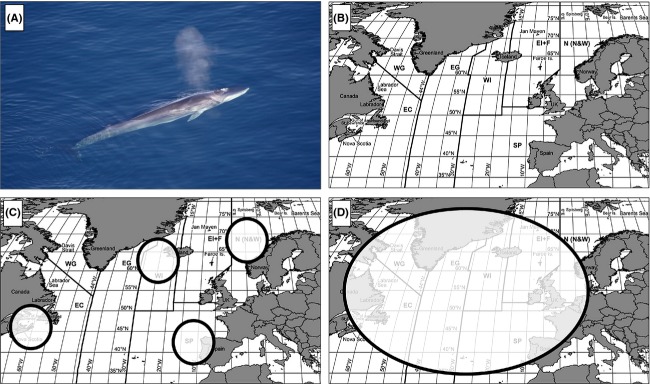
The North Atlantic fin whale. (A) adult fin whale foraging off Greenland, September 2005. (B) Map showing the delineations used by IWC to define different fin whale feeding aggregations (EC, Eastern Canada plus the Eastern USA; WG, West Greenland; EG, East Greenland; WI, West Iceland; EI+F, East Iceland and Faroe Islands; N, North and West Norway; SP, Spain). (C) schematic representation of the fin whale population structure suggested by the analysis of enzyme loci (Daníelsdóttir et al. 1991, 1992). (D) schematic representation of the fin whale population structure suggested by the analysis of microsatellite markers (Bérubé et al. 1998). Photo in (A) by Visit Greenland. File downloaded from Wikimedia Commons under the Creative Commons Attribution 2.0 Generic license (http://commons.wikimedia.org/wiki/File:Finhval.jpg). Maps in (B)–(D) modified from IWC (2009).

Fin whale liver and muscle tissue samples were collected by biologists during commercial whaling operations undertaken off western Iceland and Spain in the period 1985–1989 (Fig. 1; Table 1). Allozyme data were collected from liver samples of 327 individual fin whales, as detailed in the previous allozyme study (Daníelsdóttir et al. 1991). STR data were collected from genomic DNA extracted from muscle tissue samples from a total of 400 individuals. Included in these two datasets were 115 individuals from which both allozyme data and STR genotypes were available (i.e., both a liver and a muscle sample had been collected). In addition, we sequenced all exons in the DNA encoding the cytosolic malate dehydrogenase 1 (*MDH-1*) and mannose-6-phosphate isomerase (*MPI*) allozymes in a total of 34 animals from Iceland. Each of these individuals had known allozyme electromorphs and were selected to ensure an equal representation of each *MDH-1* and *MPI* allozyme electromorph.

**Table 1:** Number of North Atlantic fin whale samples analyzed for each genetic marker

Locality	Year	Number of samples per type of genetic marker				

		Allozyme	Microsatellite	Overlap	Combined	MDH-1 and MPI exons
Spain (ESP)	1985	46	43	42	47	
Iceland (IC)	1983		124		124	
	1985	65	158	65	158	18
	1986	71			71	9
	1987	77	9	8	78	2
	1988	68			68	5
	1989		66		66	
Sum		327	400	115	612	34

### Experimental methods

#### Allozyme and STR genotyping

The experimental conditions used to generate the allozyme data are described in the study by Daníelsdóttir et al. (1991) (Table S1). Genomic DNA for STR genotyping was extracted using 15% Chelex 100 Resin (Bio-Rad Inc.) and Proteinase K as outlined by Walsh et al. (1991). The STR loci were amplified as detailed in Table S2 (Valsecchi and Amos 1996; Palsbøll et al. 1997; Bérubé et al. 2000). All polymerase chain reactions (PCR, Mullis and Faloona 1987) were performed in a total volume of 10 *μ*L, including 2 *μ*L genomic DNA, 0.6 U DyNAzyme™ DNA polymerase (Finnzymes, Thermo Scientific, Waltham, MA, USA), 10× DNA polymerase buffer with 10 mmol/L Tris-HCl, 10 mmol/L KCl, 1.5 mmol/L MgCl_2_ and 0.1% Triton X-100 (Finnzymes, Thermo Scientific, Waltham, MA, USA), 8 *μ*mol/L dNTPs and between 0.7–4.0 *μ*mol/L of each forward and reverse primer. PCR amplifications were performed using a Thermal Cycler 225 (MJ Research Inc., St. Bruno, Canada) with 4 min at 94 degrees celsius (°C) followed by 32–35 cycles of each 50 sec at 94°C, 50 sec at 54 to 64°C, and 90 sec at 72°C, and finally a single cycle of 7 min at 72°C. PCR amplification products were separated on an ABI3730 DNA Analyzer™, sized using a GeneScan™ – 500LIZ size standard (Applied Biosystems Inc., Waltham, MA, USA). STR alleles were scored manually using the GeneMapper™ Analysis Software version 4.0 (Applied Biosystems Inc.).

#### DNA sequencing

DNA sequencing was performed on exons encoding *MDH-1* and *MPI* because they were the two most divergent allozyme loci (see Results). Genomic DNA was extracted using either standard phenol/chloroform extractions (Sambrook et al. 1989) or the DNeasy™ blood and tissue kit according to the manufacturer's instructions (QIAGEN Inc., Venlo, The Netherlands). Sequencing primers were designed from the alignment of *MDH-1-* and *MPI*-coding DNA sequences obtained from human (*Homo sapiens*), cow (*Bos Taurus*,), and pig (*Sus scrofa*) from the NCBI Gene database (Table S3). In addition, *MDH-1-* and *MPI*-coding DNA sequences from bottlenose dolphin (*Tursiops truncatus*) were obtained by a BLAST search (Altschul et al. 1990) in the NCBI Sequence Read and Trace Archive using the human *MDH-1* and *MPI* DNA sequences. Sequence alignments were performed in Geneious™ v. 5.4 (Drummond et al. 2011) using a global alignment with free end-gaps, a 65% similarity cost matrix, a gap open penalty of 10,000, and a gap extension penalty of 10,000 in the Geneious™ alignment algorithm. Initial *in silico* evaluation of primer performance was conducted using AmplifX v. 1.5.4 (Jullien 2008). When possible, primer pairs were placed in conserved regions in the introns flanking the targeted exons. In some cases, flanking intron sequences were insufficiently conserved in the alignment of NCBI sequences, necessitating the design of primers in the exon to sequence the flanking intron in a small panel of fin whale samples. The fin whale–specific intron sequences obtained in this manner were then subsequently employed as the basis for designing primers for sequencing the exons. PCR conditions consisted of 2 min at 94°C, followed by between 29–35 cycles at 94°C for 30 sec, at 54–60°C for 30 sec, and finally at 72°C for 45–74 sec followed by a single cycle at 72°C for 10 min (Table S4). PCR products were purified by shrimp alkaline exonuclease digestion (Werle et al. 1994) and sequenced using the forward or reverse primers used in the initial PCR, and the ABI BigDye™ Terminator Cycle Sequencing Kit v3.1 (Applied Biosystems Inc.) according to the manufacturer's protocol. The order of sequencing fragments was resolved on an ABI 3130 Genetic Analyzer™ (Applied Biosystems Inc.), and chromatograms were aligned and manually edited in Geneious™ (v. 5.4, Drummond et al. 2011) using the corresponding human exon sequences as reference. As control, the 11 sequence loci containing single-nucleotide polymorphisms (SNPs) were re-amplified and resequenced in on average 21% (*n* = 7) of the individuals. In addition, to assess the authenticity of our DNA sequence data, we mapped them to the recently published minke whale (*Balaenoptera acutorostrata*) genome (Yim et al. 2014) using a BLAST search in the whole-genome shotgun database.

### Data analysis

#### Genetic divergence at allozyme and STR loci

Input files for statistical analyses of the allozyme and microsatellite data were created using CONVERT ver 1.31 (Glaubitz 2004). Observed (*H*_O_) and expected heterozygosity (*H*_E_), Weir and Cockerham's (1984) *F*-statistics with 95% confidence intervals, and the deviation from Hardy–Weinberg expectations as well as linkage equilibrium was estimated for each locus and all data combined using the FSTAT package (ver 2.9.3.2, Goudet 1995). Pairwise *F*_ST_ values (and their 95% bootstrap confidence intervals) between sampling areas and/or years were estimated using FSTAT. We used FDIST2 (Beaumont and Nichols 1996) to identify outlier loci (inferred from the degree of genetic divergence estimated as *F*_ST_) implemented in LOSITAN (Antao et al. 2008), assuming an infinite allele model for the allozyme data and a stepwise mutation model for the STR data. We employed the options “Neutral mean *F*_ST_” and “Neutral+Forced mean *F*_ST_” with 100,000 iterations, a 99% confidence interval, a false discovery rate of 1%, and a subsample size at 50.

#### Nucleotide substitutions in the *MDH-1* and *MPI DNA* sequences

SNPs in the *MDH-1* and *MPI* sequences of the fin whale were identified as single-nucleotide differences either in the homozygote or in the heterozygote state. The frequencies of each SNP variant as well as the observed and expected heterozygosity were determined using SNPator (Morcillo-Suarez et al. 2008). Pairwise tests of linkage disequilibrium were performed using GENEPOP v. 4.0 (Rousset 2008) and significance assessed using the sequential Bonferroni correction (Holm 1979). We used ARLEQUIN (Excoffier and Lischer 2010) to estimate the sequence-level polymorphism, *θ* (Watterson 1975)*,* and average nucleotide diversity, *π* (Nei 1987)*,* for the concatenated exon sequences only, as well as for exons and partial intron sequences combined.

#### Inferred amino acid variation in *MDH-1* and *MPI*

The DNA sequences of the *MDH-1-* and *MPI*-coding regions were translated into the corresponding amino acid sequences to identify synonymous and nonsynonymous nucleotide substitutions. To examine homology and provide an additional indication of the authenticity of our inferred fin whale protein sequences, these were aligned and compared with the equivalent *MDH-1* and *MPI* protein sequences from human, cow, pig, rat (*Rattus norvegicus*), and dog (*Canis lupus familiaris*) obtained from the NCBI GenBank database (http://www.ncbi.nlm.nih.gov/genbank) and UniProtKB database (http://www.unitprot.org) (Table S3). Translations and alignments were performed in Geneious v. 5.4 (Drummond et al. 2011) using a global alignment with free end-gaps, a Blosum62 cost matrix, a gap open penalty of 12 and gap extension penalty of 3.

#### Alternative factors causing electrophoretic variation in allozyme loci?

In order to assess potential alternative factors causing electrophoretic variation in the fin whale *MDH-1* and *MPI* allozyme loci we performed additional assessments of experimental artifacts, alternative splicing and post-translational modifications (PTMs).

First, to explore potential experimental artifacts caused by sample storage the strength and statistical significance of the correlation between allozyme electromorph frequencies and sampling year was estimated by linear regression and an *F*-test as implemented in the Microsoft Excel Analysis ToolPak (Microsoft Inc.).

Second, in the absence of fin whale reference data and given the logistical and ethical difficulties associated with obtaining new high-quality fin whale tissue samples for laboratory testing, we extracted information about active sites, putative splice forms (isoforms), and PTMs in human, mouse, and rat from the UniProtKB, PhosphositePlus (Hornbeck et al. 2004), and PHOSIDA (Gnad et al. 2011) databases, as well as a novel atlas of tissue-specific phosphorylation in the mouse (Huttlin et al. 2010). Next, we used this information on known *MDH-1* and *MPI* protein isoforms in human, mouse, and rat to infer putative protein isoforms in fin whales. The molecular weight, isoelectric point, net electric charge, and instability index of putative isoforms in the fin whale was estimated using the package ProtParam (Gasteiger et al. 2005). In addition, *in silico* prediction of putative PTM sites for acetylation, phosphorylation, and sumoylation in the fin whale *MDH-1* and *MPI* proteins was performed using NetAcet (Kiemer et al. 2005), NetPhos (Blom et al. 1999), and SUMOsp (Ren et al. 2009), respectively. To reduce the frequency of false positives, we applied the most conservative cutoff values (i.e., “high”) for each of the estimations.

Finally, as the allozyme variation observed at *MDH-*1 and *MPI* may be a response to metabolic processes (Slein 1950; Gracy and Noltmann 1968; Proudfoot et al. 1994), we employed linear regression, ANOVA, and Student's *t*-tests implemented in the Microsoft Excel Analysis ToolPak (Microsoft Inc.) to assess the strength and statistical significance of potential correlations in allozyme electromorph frequencies with fin whale body condition. Morphological data for the fin whales included in the allozyme study was obtained from Víkingsson (1990). Estimates of half girth-width, blubber thickness, and total body length, were converted into measures of blubber thickness/body length and half girth-width/body length, respectively, as a measure of the relative body condition of each individual fin whale.

## Results

### Genetic divergence at allozyme and STR loci

Ten of the 40 enzyme loci screened for allozymes by Daníelsdóttir et al. (1991) and 15 of the STRs yielded consistent and polymorphic genotypes in the majority of fin whale samples (Tables S1 and3). As expected, the estimates of genetic diversities were higher for STR loci (*H*_O_ = 0.77, 95% CI = 0.73–0.81; *H*_E_ = 0.80, 95% CI = 0.76–0.83) than allozyme loci (*H*_O_ = 0.259, 95% CI = 0.160–0.358; *H*_E_ = 0.332, 95%CI = 0.212–0.452). Several allozyme (*n* = 6) and STR (*n* = 5) loci exhibited a statistically significant degree of heterozygote deficiency and a single STR loci exhibited heterozygote excess. None of the microsatellite and allozyme loci exhibited significant linkage disequilibrium after sequential Bonferroni correction.

Among the allozyme loci, the degree of genetic divergence between sample areas was moderate to high with *F*_ST_ = 0.028–0.197 and significantly different from zero all estimations (Table 2). In contrast, the degree of genetic divergence between sampling areas at the STR loci was low. Estimates of *F*_ST_ ranged from zero to 0.0008 and did not differ significantly from zero in any of the tests (Table 3). The overall degree of genetic divergence was significantly higher (two-tailed sign test, *P* < 0.002) at the allozyme loci (*F*_ST_ = 0.103, 95% CI = 0.049–0.165) than at the STR loci (*F*_ST_ < 0.001, 95% CI = 0.000–0.001).

**Table 2:** Estimates of genetic differentiation at 10 allozyme loci among the five sampling groups of North Atlantic fin whales

	ESP85	IC85	IC86	IC87	IC88
ESP85		0.1206	0.1149	0.1984	0.1972
IC85	0.015–0.231		0.0470	0.1101	0.1625
IC86	0.029–0.203	0.003–0.082		0.0280	0.0644
IC87	0.050–0.345	0.031–0.178	0.009–0.049		0.0634
IC88	0.046–0.373	0.052–0.267	0.025–0.109	0.016–0.113	

Pairwise *F*_ST_ estimates above diagonal (Weir and Cockerham 1984); 95% bootstrap confidence interval below diagonal.

**Table 3:** Estimates of genetic differentiation at 15 microsatellite loci among the five sampling groups of North Atlantic fin whales

	ESP85	IC83	IC85	IC87	IC89
ESP85		0.0009	0.0008	0.0000	0.0000
IC83	0–0.004		0.0000	0.0000	0.0000
IC85	0–0.005	0–0.001		0.0000	0.0000
IC87	0–0.013	0–0.003	0–0.004		0.0000
IC89	0–0.002	0–0.001	0–0.001	0–0.001	

Pairwise *F*_ST_ estimates above diagonal (Weir and Cockerham 1984); 95% bootstrap confidence interval below diagonal.

**Figure 2:**
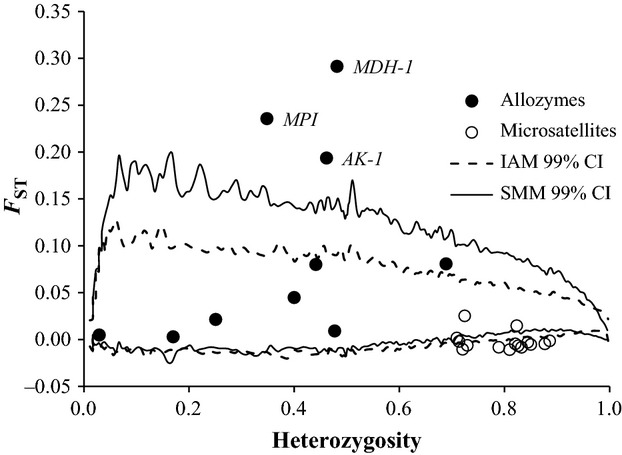
Detection of outlier loci using the FDIST2 (Beaumont and Nichols 1996) method implemented in LOSITAN (Antao et al. 2008). Loci above the 99% confidence intervals have higher than expected *F*_ST_ values and are candidates for being under divergent selection. Loci below the 99% confidence intervals have lower than expected *F*_ST_ values and are candidates for being under balancing selection. Filled circles = allozyme loci; open circles = microsatellite loci; full line = the 99% confidence interval under the stepwise mutation model; stippled line = the 99% confidence interval under the infinite alleles mutation model. The three enzyme loci *MDH-1*, *MPI*, and *AK-1* in North Atlantic fin whales were consistently identified as *F*_ST_ -outliers.

In the outlier test, the width of the 99% CIs varied slightly depending upon which mutation model was assumed (infinite alleles vs. stepwise mutation) and the choice of simulation model (“Neutral mean *F*_ST_” vs. “Neutral + Forced mean *F*_ST_”) as well as whether the allozyme and STR data were analyzed together or separately. However, three allozyme loci *MDH-1*, *MPI*, and *AK-1* were consistently identified as outlier loci with above average *F*_ST_ values, suggesting the possibility of divergent selection (Fig. 2). Eleven of the 15 STR loci had lower than average *F*_ST_'s in estimations including allozyme and STR data; however, this pattern was not observed when only STR data were analyzed.

### Nucleotide substitutions in the *MDH-1* and *MPI* DNA sequences

**Figure 3:**
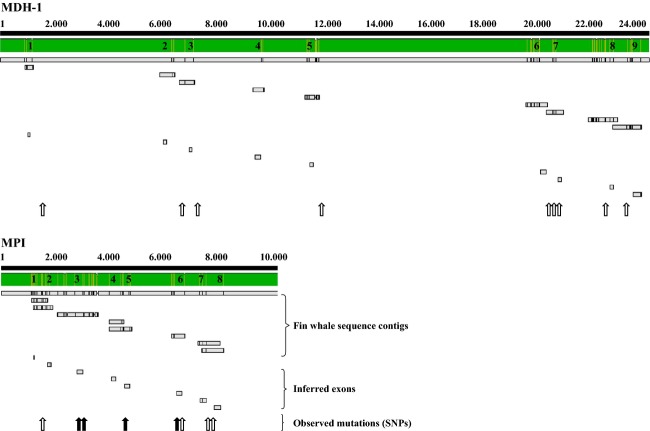
The inferred fin whale *MDH-1* and *MPI* genes mapped to the minke whale genome. For each gene, the fin whale sequence contigs, the inferred exons and the approximate location of observed mutations are listed. Open arrows = synonymous mutations in introns; black arrows = synonymous mutations in exons. No nonsynonymous mutations were observed. The minke whale whole-genome sequences have accession numbers ATDI01127815.1 and ATDI01127816.1 for *MDH-1* and ATDI01006327.1 for *MPI*.

We designed 18 primer pairs to sequence the *MDH-1* and *MPI* exons and partial introns (Table 4 and Table S4). Each of the inferred fin whale *MDH-1* and *MPI* exons mapped to a single location on the minke whale genome, strongly suggesting that we sequenced the correct genes (Fig. 3). The total sequence coverage was more than 11,000 base pairs (bp), but bidirectional coverage in at least 95% of the animals were only obtained for 3300 bps of the *MPI* gene and 3908 bp of the *MDH-1* gene. In these regions, a total of 18 SNPs were identified. Nine SNPs were detected in the *MPI* gene; five SNPs were located in the introns and four in the exons. Nine SNPs were also detected in the *MDH-1* gene, all of which were located in the introns. Two SNPs located in the introns of *MDH-1* and *MPI*, respectively, could not be consistently genotyped and were therefore omitted from further analysis. As genotyping control, on average, 21% of the individuals were resequenced per sequence locus, revealing a single mismatch in a SNP and thus genotyping error-rate below 1%. There was no correlation between genotyping success and allozyme phenotype (data not shown).

**Table 4:** Characteristics of the 18 single-nucleotide polymorphisms (SNPs) detected in the *MDH-1* and *MPI* genes of the fin whale. A SNP in intron 6 of the *MPI* gene (*MPI*6-367) and a SNP in intron 8 of *MDH-1* (MDH9-022) could not be genotyped consistently and were omitted from further analyses

Gene	SnpID	PCR locus	Locus position	Hs region	Hs position	Alleles	*N*	MAF	*H*_O_	*H*_E_	*P*
*MDH-1*	MDH1-184	Mdh1-1	184	Intron 1	184	A/G	34	0.029	0.059	0.057	0.965
	MDH2-393	Mdh1-2	393	Intron 2	5873	C/T	34	0.147	0.235	0.251	0.834
	MDH5-418	Mdh1-5	418	Intron 5	10564	A/G	34	0.074	0.147	0.136	0.854
	MDH6-257	Mdh1-6	257	Intron 6	15505	A/G	34	0.191	0.324	0.309	0.857
	MDH6-333	Mdh1-6	333	Intron 6	15581	A/C	34	0.029	0.059	0.057	0.965
	MDH6-562	Mdh1-6	562	Intron 6	15817	A/T	34	0.029	0.059	0.057	0.965
	MDH6-563	Mdh1-6	563	Intron 6	15818	A/T	34	0.015	0.029	0.029	0.988
	MDH8-345	Mdh1-8	345	Intron 8	17312	C/T	33	0.015	0.030	0.030	0.988
	MDH9-022	Mdh1-9	22	Intron 8	17788	C/T	34	NA			
*MPI*	MPI1-161	MPI-1	161	Intron 1	76	C/T	34	0.353	0.294	0.457	0.057
	MPI3-280	MPI-3	280	Exon 3	1469	C/T	34	0.044	0.088	0.084	0.935
	MPI3-296	MPI-3	296	Exon 3	1485	C/T	34	0.044	0.088	0.084	0.935
	MPI5-397	MPI-5	397	Exon 5	3246	C/G	34	0.441	0.353	0.493	0.102
	MPI6-326	MPI-6	420	Exon 6	6234	C/T	33	0.046	0.030	0.087	0.249
	MPI6-367	MPI-6	367	Intron 6	6275	G/T	33	NA			
	MPI6-420	MPI-6	368	Intron 6	6328	A/G	34	0.059	0.118	0.111	0.898
	MPI78-456	MPI-78	456	Intron 7	7347	C/T	34	0.059	0.118	0.111	0.898
	MPI78-486	MPI-78	486	Intron 7	7386	A/G	34	0.015	0.029	0.029	0.988

PCR Locus, the PCR locus referred to in Table S4; Hs Region, regional location in the human gene (*MDH-1* gene ID: 154200; *MPI* gene ID: 4351); Hs Position, position in the human gene; *N,* number of samples genotyped; MAF, minor allele frequency; *H*_O_, observed heterozygosity; *H*_E_, expected heterozygosity; *P,* probability of the SNP being in Hardy–Weinberg equilibrium; NA, not analyzed because of genotyping uncertainties.

No statistically significant deviations from the expected Hardy–Weinberg genotype frequencies were observed for any SNP. In the *MPI* gene, four of the 28 pairwise tests of linkage disequilibrium among SNPs were statistically significant at the 5% level after sequential Bonferroni correction, and two of the 36 pairwise linkage disequilibrium tests were significant at the 5% level in *MDH-1* gene. All nonsignificant tests were between SNPs with minor allele frequencies (i. e., <5%), suggesting that the lack of significant LD within genes could be due to small sample sizes (i.e., few gene copies of the rare SNP allele), rather than recombination. We did not detect linkage between SNPs located on different genes. The average nucleotide diversity *π* and level of polymorphism *θ* was low for the exons and partial introns of *MPI* (*π* = 0.0005; *θ* = 0.0008), for the *MPI* exons alone (*π* = 0.0006; *θ* = 0.0013) and for the exons and partial introns of the *MDH-1* gene (*π* = 0.0002; *θ* = 0.0007) and was zero for the *MDH-1* exons alone (which did not contain SNPs).

### Inferred amino acid variation in *MDH-1* and *MPI*

The amino acid sequences inferred from the fin whale *MDH-1-* and *MPI*-coding DNA sequences were similar to the annotated amino acid sequences from other mammals. The pairwise identity scores averaged 96% and 88% for inferred *MDH-1* and *MPI* amino acid sequences, respectively (Figs. S1 and S2). Interestingly, as no SNPs were detected in the exons of the *MDH-1* gene and all SNPs located in the exons of the fin whale *MPI* gene were synonymous substitutions, our DNA sequence data did not indicate variation in the fin whale *MDH-1* and *MPI* proteins. That is, the nucleotide sequences obtained from the exons coding the *MDH-1* and *MPI* allozymes appeared incompatible with the previously reported allozyme variation and differentiation among North Atlantic fin whales.

### Alternative factors causing electrophoretic variation in allozyme loci?

**Figure 4:**
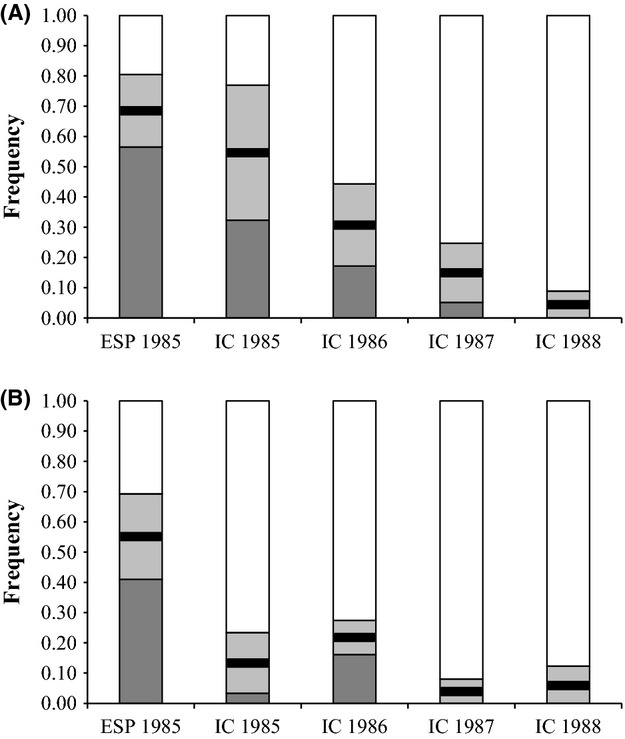
Changes in electromorph and genotype frequencies at the *MDH-1* (A) and *MPI* (B) allozyme loci for Spanish (ESP) and Icelandic (IC) samples obtained in 1985-1988. White = homozygotes in the slow least negatively charged electromorph; light gray = heterozygotes; dark gray = homozygotes of the fast most negatively charged electromorph. The black bars denote the frequency of the fast electromorph and the trend line the correlation between these frequencies and sampling locality/year for *MDH-1* with (*Y* = −0.168*X* + 0.850, *R*^2^ = 0.984, *F* = 186.19, *P* = 0.0009) and without (*R*^2^ = 0.968, *F* = 61.29, *P* < 0.0001) the Spanish samples. The corresponding figures for *MPI* were (*R*^2^ = 0.669, *F* = 6.07, *P* = 0.0905) and (*R*^2^ = 0.409, *F* = 1.38, *P* = 0.3606), respectively.

The assessment of potential effects of sample storage upon allozyme variation revealed that the frequencies of the two allozyme electromorphs detected at the *MDH-1* locus were strongly and highly significantly correlated with sampling year among the Icelandic fin whale samples. The frequency of the most negatively charged *MDH-1* allozyme electromorph decreased at a rate at approximately 17% per year (Fig. 4A). In contrast, the change in *MPI* allozyme electromorph frequencies did not correlate with time (Fig. 4B).

In our *in silico* inference of likely alternative splicing we found three known isoforms of the human *MDH-1* enzyme, here denoted I-III (Table S5). Isoform *III* appears to be specific to humans and was disregarded in the subsequent analyses. To examine whether the two fin whale electromorphs reported in the allozyme study may correspond to the remaining *MDH-1* isoforms I and II observed in humans, we estimated their electrical charge of the putative *MDH-1* isoforms I and II using the inferred fin whale amino acid sequence. A putative fin whale isoform I was inferred which corresponded to the human isoform *II* and which would carry a net negative charge, whereas a putative isoform II would carry a net positive charge in fin whales, suggesting that the two isoforms would migrate in different directions on a polyacrylamide gel. As this contrasts with the electrophoretic pattern reported for the slow and fast *MDH-1* electromorphs observed in the allozyme study (Fig. S3), we assume that those are different from the human isoforms II and III. The human *MPI* protein exists in four known isoforms denoted I-IV. Again assuming that the above isoforms occur in fin whales, we found that all isoforms carries a net negative charge and hence could be the slow and fast electromorphs observed in the allozyme study (Fig. S3). Thus, we did not find support for alternative splicing in fin whale *MDH-1*, but it could occur in *MPI*.

We identified 18 *MDH-1* amino acid residues that were known targets of PTM in human, mouse, and/or rat, and an additional four inferred amino acid residues were predicted *in silico* as PTM sites using NetAcet, NetPhos, and SUMOsp (Table S6). Two PTM sites are known in human, mouse, and/or rat *MPI*, and the NetPhos and SUMOsp approaches inferred additional 10 nucleotide sites based on the inferred fin whale *MPI* amino acid sequence. In *MDH-1*, one of the 22 known or predicted PTM sites appeared variable across different mammal species, whereas five of the 12 sites in *MPI* were variable (Figs. S1, S2 and Table S6). Of these, residue 332 of *MDH-1* was alanine and a putative acetylation site in fin whale, bottlenose dolphin and cow, but a serine and potential phosphorylation site in human, rat, dog, and pig. Also, residue 389 of *MPI* was serine and predicted phosphorylation site in fin whale, humpback whale and dolphin, but proline in human, rat, dog, pig, and cow. These two sites may be subject to post-translational modifications in fin whales.

**Figure 5:**
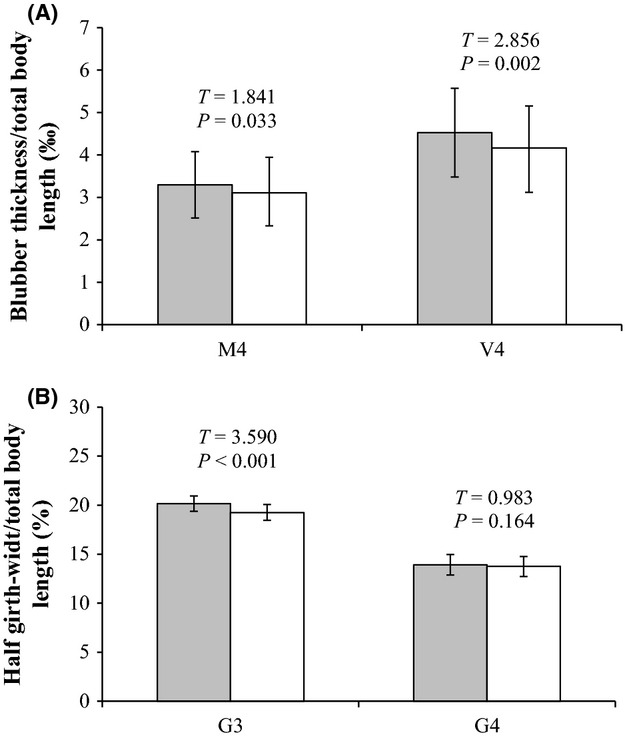
Comparisons of average body condition for fin whales with different *MDH-1* allozyme genotypes. Gray bars are fin whales with FF or FS allozyme genotypes (i.e., those carrying the fast electromorph) and white bars are individuals with the SS genotype (those without the fast electromorph), with the standard deviation marked as error bars. (A) Blubber thickness in ‰ body length measured in on the side (M4) or ventrally (V4) just in front of the dorsal fin. (B) Half girth-width in% body length measured on halfway between the pectoral and dorsal fins (G3) or just in front of the dorsal fin (G4). Statistical significance was assessed by a *t*-test.

Finally, in assessing the potential correlation between allozyme electromorphs and fin whale body condition, we found that the index of relative body condition in individual fin whales was significantly higher in animals that carried the fast *MDH-1* electromorph in homozygote or heterozygote state compared with animals that did not carry this electromorph (Fig. 5A and B), suggesting that this electromorph may be associated with a metabolic process in fin whales. No such patterns were detected for the *MPI* allozyme electromorphs.

## Discussion

Our combined analysis of 10 allozyme and 15 STR loci in North Atlantic fin whales identified three outlier allozyme loci *MDH-1*, *MPI*, and *AK-1*, all of which exhibited well above average levels of genetic divergence among sampling years and localities. However, when sequencing the exons of the two most divergent allozyme loci, *MDH-1* and *MPI*, we only identified four synonymous nucleotide substitutions and no nonsynonymous substitutions. In itself, the low level of genetic polymorphisms in these nuclear loci is consistent with other studies of baleen whales (Palumbi and Baker 1994; Gaines et al. 2005; Jackson et al. 2009), but the observed absence of nonsynonymous substitutions is in contrast with the generally applied notion that allozyme variation is governed by nucleotide substitutions in the underlying coding DNA sequence (Kreitman 1983; Griffith and Powell 1997; Fields and Somero 1998; Hasson et al. 1998; Pogson 2001; Protas et al. 2006; Wheat et al. 2006, 2010; Linnen et al. 2009; McCracken et al. 2009a,b2009b; Storz et al. 2009; Crease et al. 2011; Scott et al. 2011; Schoville et al. 2012). Still, our findings may not be an uncommon phenomenon in natural populations as the majority of previous reports were based upon readily observable selective agents and differences in phenotype (Griffith and Powell 1997; Fields and Somero 1998; Protas et al. 2006; Wheat et al. 2006, 2010; McCracken et al. 2009a,b2009b; Storz et al. 2009; Crease et al. 2011; Scott et al. 2011) and thus potentially biased toward organisms and genes with clear links between phenotype and genotype. In contrast, natural populations and species typically do not exhibit clear phenotypic differences, may be subject to weaker and cryptic selective agents and/or are difficult to study because of their elusive nature, suggesting that observations like ours may be underrepresented, or unreported. The question is what governs the observed allozyme variation in fin whales if not nonsynonymous substitutions?

### Experimental artifacts?

It is well known that experimental artifacts may result in the detection of false polymorphisms in analyses of allozyme loci (May 1998). In our analyses of the allozyme data, we made two observations, which could suggest that the polymorphisms reported for the *MDH-1* and *MPI* allozyme loci result from such experimental artifacts. First; the *MDH-1* locus exhibited a gradual change in allozyme electromorph frequencies across sampling years, a pattern often associated with experimental bias. For example, similar to *MDH-1*, the alcohol dehydrogenase (*ADH*) enzyme contains several binding sites for the coenzyme nicotinamide adenine dinucleotide (NAD), which, as a consequence of suboptimal storage conditions, can dissociate from *ADH*, thereby changing the enzymes' electrophoretic mobility, which may be incorrectly inferred as allozyme variation (McKinley and Moss 1965; Jacobson 1968; Lakovaara and Saura 1970). Second; in contrast with the *MDH-1* and *MPI* enzyme, polymorphisms reported in the fin whale allozyme studies (Daníelsdóttir et al. 1991, 1992), the *MDH-1* and *MPI* enzyme loci were found to nonvariable in the majority of more than 15,500 samples screened in other allozyme studies of baleen and toothed whales (Table S7) (Simonsen et al. 1982b; Wada 1983a,b1983b, 1988; Shimura and Numachi 1987; Andersen 1988; Wada and Numachi 1991).

There are, however, also several factors speaking against experimental artifacts. First, in the fin whale study from which the allozyme data came, several precautionary steps were taken to avoid experimental artifacts such as sampling, storage, handling, and analysis (Daníelsdóttir et al. 1991, 1992; Daníelsdóttir 1994). Second, the *MDH-1* electromorph frequencies did not change with time in samples collected from sei whales (*Balaenoptera borealis*), which were processed simultaneously with the fin whale samples (Daníelsdóttir et al. 1991). Third, the nonvariable *MDH-1* and *MPI* enzyme loci reported in other studies and species could result from the use of starch gel electrophoresis, which has a lower resolution compared with the polyacrylamide gels used to generate the fin whale allozyme data reported here (Daníelsdóttir et al. 1991). Such “hidden” polymorphism owing to the use of different electrophoretic conditions is common among allozyme studies (Bernstein et al. 1973; Cochrane 1976; Coyne 1976, 1982). Finally, artifacts resulting from us sequencing the incorrect DNA regions seems unlikely as our fin whale *MHD-1* and *MPI* DNA sequences each mapped to a single region of the recently published minke whale genome. Also, we observed a large degree of similarity between our inferred exon and protein sequences and the publically available *MDH-1* and *MPI* exon and protein sequences obtained from other mammals. Hence, there is little to suggest that experimental artifacts account for the discrepancy between *MDH-1* and *MPI* enzyme- and DNA-level variation, although the possibility cannot be completely ruled out.

### Alternative splicing and post-translational modifications?

A plausible explanation for the observed discrepancy between enzyme- and DNA-level variation involves alternative splicing and post-translational modifications (King and Wilson 1975; Matlin et al. 2005; Marden 2008; Chen and Manley 2009; Keren et al. 2010; Kelemen et al. 2013). In our assessment of the fin whale *MDH-1* and *MPI* enzymes, we assumed homology to the corresponding proteins in human, mouse, and rat and found no indication of alternative splicing in fin whale *MDH-1*, a finding that agrees with preliminary fin whale transcriptome data (Per Palsbøll, unpublished). In contrast, we cannot rule out alternative splicing as a cause for the observed *MPI* enzyme polymorphisms.

PTMs may result in several differently charged or folded states of the protein through enzyme-catalyzed modifications of the side chains or backbones of the folded protein (Walsh et al. 2005). Apparent polymorphisms in enzyme loci due to PTMs is a well-known phenomenon (Harris and Hopkinson 1976) and have previously been inferred as the cause of false parentage analyses in sparrows (Wetton et al. 1992) and non-Mendelian inheritance in fish (Crozier and Moffett 1990). The *MDH-1* and *MPI* enzymes contains several residues that are known targets of PTM in human, mouse, and rat or were inferred from *in silico* analysis of the fin whale primary protein sequence. Two of these PTM sites appeared specific to fin whales (Table S6). In fact, previous electrophoretic screening of the *MDH-1* enzyme locus found that different tissues from individual fin whales had different electromorph phenotypes (Daníelsdóttir 1994), which is indicative of PTMs in this locus. This pattern was not observed for *MPI*.

### Function of *MDH-1* and *MPI* in a biological context?

Assuming that the *MDH-1* and *MPI* genes indeed are post-translational modified and alternatively spliced, respectively, in fin whales, are there any characteristics of their cellular function and the biology of fin whales that may provide a clue as to why? North Atlantic fin whales are believed to undertake seasonal movements between feeding and breeding areas (Rørvik and Jonsgård 1981; Donovan 1991; Víkingsson et al. 2009). Fin whales are filter feeders, preying primarily on zooplankton (e.g., euphasiids) to build up adequate fat storages for periods with limited nutritional intake (Hinga 1979; Lockyer 1986, 2007; Víkingsson 1990, 1997). Measurements of blubber thickness and girth-width in the period 1975–1988 document annual variations in fin whale body condition and female fecundity (Lockyer 1986, 2007; Víkingsson 1990), correlating with similar variations in zooplankton biomass (Beare et al. 2000; Lockyer 2007). The combination of high energetic requirements, a relatively short feeding season with unpredictable fluctuations in prey availability, and prolonged periods of reliance on stored lipids (Lockyer 1986, 2007; Potvin et al. 2012) could necessitate a degree of flexibility in the function of key metabolic enzymes that cannot be allowed for by amino acid substitutions, but could be obtained by cis-regulatory processes, such as alternative splicing and PTMs.

Indeed, several studies have documented the role of PTMs in regulating the activity of metabolic enzymes such as *MDH-1* in response to cellular demands (Choudhary et al. 2009; Wang et al. 2010; Zhao et al. 2010). In a recent study, Kim et al. (2012) found that increased acetylation of *MDH-1* during adipogenesis dramatically enhanced its enzymatic activity. They proposed that this activity supports acetyl coenzyme A (acetyl-CoA) and *NADPH* in lipid synthesis by accelerating the citrate shuttle and that *MDH-1* performs a key function as cross-talk mechanism between lipid synthesis and intracellular energy levels (Kim et al. 2012).

The observed differences in relative body condition index of fin whale individuals with and without the fast *MDH-1* electromorph does point to a link between *MDH-1* and fin whale body condition. More specifically, given: (1) reported decreases in zooplankton biomass (Beare et al. 2000) and in fin whale lipid content in the period 1985–1988 (Víkingsson 1990); (2) our findings that the frequency of the *MDH-1* fast electromorph decreased during that same period (Fig. 4A); and (3) that the absence of this electromorph was associated with significantly reduced body condition (Fig. 5A–B), we tentatively propose that the fast *MDH-1* electromorph result from acetylation of *MDH-1* and that its observed decreasing frequency is associated reduced lipid synthesis as a result of limited prey availability.

### Implications for the study of natural populations

Regardless of the causative agent, the observed discrepancy between enzyme- and DNA-level variation has important implications for the study of selection and adaptation in natural populations, and for the general use of allozyme markers in population genetic studies. The detection of allozyme, microsatellite, or SNP loci deviating from neutral expectations has often been inferred as evidence for selection in the marker itself or in closely linked genes, and consequently local adaptation (e.g., Eanes 1999; Lemaire et al. 2000; Pogson and Fevolden 2003; Hemmer-Hansen et al. 2007; Larsson et al. 2007; Skarstein et al. 2007; Nielsen et al. 2009b; White et al. 2010; Andre et al. 2011; Kirk and Freeland 2011; Richter-Boix et al. 2011; Chaoui et al. 2012). Our findings stress that deviations from neutral patterns in outlier loci does not imply that such loci are under selection, and if the deviation from neutrality indeed is of biological significance, the underlying mechanisms may be governed by a complex, but more flexible, interplay of protein-coding and cis-regulatory processes. This may in particular concern allozyme loci, which were the markers of choice for several decades (e.g., Bonnell and Selander 1974; Ferguson and Mason 1981; Simonsen et al. 1982a; O'Brien et al. 1983) and still find their use in population genetic studies (Clarke and Whyte 2003; Toda et al. 2003; Curole et al. 2004; Vuorinen and Eskelinen 2005; Matsui et al. 2006; Larsson et al. 2007; Addison et al. 2008; Silva and Skibinski 2009; Andre et al. 2011; Chaturvedi et al. 2011; Crease et al. 2011; Pinho et al. 2011; Sa-Pinto et al. 2012; Strand et al. 2012). The data presented here suggest that reports of outlier loci should be interpreted with great caution. Inferred levels of genetic divergence and polymorphisms may not be the product of random genetic drift, migration or even selection. For this reason, it is advisable to explore the molecular background before making any conclusive inference about population structuring, migration rates, demographic history, and local adaptation from the spatial and temporal distribution of allozyme variation.

Finally, with regard to the North Atlantic fin whale, our findings imply that the population structure inferred by previous allozyme studies (Daníelsdóttir et al. 1991, 1992; Daníelsdóttir 1994) should be disregarded in future assessments of the population's management status. Rather, such assessments will require much larger sample sets and number of nuclear genetic markers, as well as the adoption of novel analytical approaches (Økland et al. 2010; Palsbøll et al. 2010) to facilitate the discrimination between recent divergence and high gene flow, both of which are consistent with the low levels of population divergence reported for mtDNA and microsatellite loci (Bérubé et al. 1998).

## Supporting Information

**Table S1.** Characteristics of the previously published 10 polymorphic enzyme loci in North Atlantic fin whales that were statistically re-analyzed in the present study.

**Table S2.** Characterization of the 15 microsatellite loci in North Atlantic fin whales included in the analyses.

**Table S3.** Gene and Protein IDs for selected reference species.

**Table S4.** Characteristics of the primer pairs designed to amplify and sequence the exons and partial introns of the *MDH-1* and *MPI* nuclear genes of the North Atlantic fin whale.

**Table S5.** Characteristics of putative alternative splice isoforms of fin whale *MDH-1* and *MPI*. In the three *MDH-1* isoforms I-III, isoform I is the canonical isoform and isoform III appears to be specific to humans as exon 1 is not conserved in other mammals (data not shown).

**Table S6.** Protein residues known or predicted to be targets of post-translational modifications (PTMs).

**Table S7.** *MDH-1* and *MPI* enzyme loci polymorphisms reported for other fin whale populations and cetacean species.

**Figure S1.** The fin whale *MDH-1* protein aligned with protein sequences from other mammals.

**Figure S2.** The fin whale *MPI* protein aligned with protein sequences from other mammals.

**Figure S3.** Pictures of the original gel electrophorese of the two enzyme loci, *MDH-1* (a) and *MPI* (b) loci in North Atlantic fin whales. Modified from Daníelsdóttir (1994).
