# Supplementary material for: Fin whale MDH-1 and MPI allozyme variation is not reflected in the corresponding DNA sequences
Source: Ecol Evol. 2014 Apr 16;4(10):1787–803. doi: 10.1002/ece3.1046 (PMC4063476; doi:10.1002/ece3.1046)
Supplement: Supplementary file 1 [file ece30004-1787-SD1.docx]

**SUPPORTING INFORMATION**

**MANUSCRIPT ID**

ECE-2013-01-0035

**TITLE**

Fin whale *MDH-1* and *MPI* allozyme variation is not reflected in the corresponding DNA sequences

**AUTHORS**

Morten Tange Olsen^1$^, Christophe Pampoulie^2^, Anna K. Daníelsdóttir^3^, Emmelie Lidh^1^, Martine Bérubé^1,4^, Gísli A. Víkingsson^2^, Per J. Palsbøll^1,4^

**Table S1** Characteristics of the previously published 10 polymorphic enzyme loci in North Atlantic fin whales that were statistically re-analyzed in the present study.

| Locus | Name | Buffer^a^ | Tissue | Alleles | *H*_O_ | *H*_E_ | *F*_IT_ | *F*_ST_ | *F*_IS_ |
| --- | --- | --- | --- | --- | --- | --- | --- | --- | --- |
| *Ada* | Adenosine deaminase | PAGE(15%) | liver | 4 | 0.521 | 0.634 | 0.240 | 0.083 | **0.171** |
| *Adh* | Alcohol dehydrogenase | PAGE(5%) | liver | 2 | 0.030 | 0.029 | -0.015 | 0.002 | -0.018 |
| *Ak-1* | Adenylate kinase 1 | PAGE(12%) | liver | 2 | 0.174 | 0.374 | 0.621 | 0.157 | **0.550** |
| *Ldh-A* | Lactate dehydrogenase A | PAGE(5%) | liver | 2 | 0.362 | 0.406 | 0.164 | 0.078 | 0.093 |
| *Mdh-1* | Malate dehydrogenase 1 (soluble) | PAGE(5%) | liver | 2 | 0.248 | 0.342 | 0.465 | 0.277 | **0.261** |
| *Mpi* | Mannose-6-phosphate isomerase | PAGE(12%) | liver | 3 | 0.172 | 0.267 | 0.467 | 0.205 | **0.329** |
| *Pep-A* | Peptidase A | PAGE(7%) | liver | 3 | 0.311 | 0.474 | 0.358 | 0.007 | **0.354** |
| *Pgi* | Phosphoglucose isomerase | PAGE(4%) | liver | 3 | 0.183 | 0.169 | -0.072 | 0.002 | -0.075 |
| *Pgm-1* | Phosphoglucomutase 1 | PAGE(9%) | liver | 2 | 0.211 | 0.246 | 0.168 | 0.020 | **0.151** |
| *Sod-A* | Superoxide dismutase A (soluble) | PAGE(7%) | liver | 2 | 0.377 | 0.382 | 0.056 | 0.049 | 0.008 |
| Overall |  |  |  | 25 | 0.259 | 0.332 | 0.297 | 0.103 | 0.216 |

^a^ Information obtained from Danielsdottir *et al.* (1992)

F_IS_ estimates in bold are significantly different from Hardy-Weinberg expectations

**Table S2** Characterization of the 15 microsatellite loci in North Atlantic fin whales included in the analyses.

| Locus | Mplex | T_A_ | Cyc | Alleles | *H*_O_ | *H*_E_ | *F*_IT_ | *F*_ST_ | *F*_IS_ | Ref |
| --- | --- | --- | --- | --- | --- | --- | --- | --- | --- | --- |
| EV01 | 1 | 54 | 32 | 14 | 0.781 | 0.831 | 0.053 | -0.002 | **0.055** | a |
| EV037 | 1 | 54 | 32 | 17 | 0.847 | 0.855 | -0.016 | 0.002 | -0.018 | a |
| GATA028 | 2 | 56 | 35 | 14 | 0.835 | 0.881 | 0.005 | 0.001 | 0.004 | b |
| GATA053 | 3 | 58 | 35 | 9 | 0.694 | 0.716 | 0.006 | -0.001 | 0.008 | b |
| GATA098 | 1 | 54 | 32 | 9 | 0.802 | 0.795 | -0.009 | -0.001 | -0.008 | b |
| GATA417 | 2 | 56 | 35 | 20 | 0.929 | 0.887 | -0.029 | -0.001 | **-0.028** | b |
| GGAA520 | 4 | 54 | 32 | 10 | 0.804 | 0.847 | 0.017 | -0.002 | 0.019 | b |
| GT011 | 3 | 58 | 35 | 8 | 0.812 | 0.84 | 0.036 | -0.003 | **0.039** | c |
| GT195 | 3 | 58 | 35 | 11 | 0.778 | 0.812 | 0.002 | -0.001 | 0.003 | d |
| GT203 | 1 | 54 | 32 | 13 | 0.759 | 0.819 | 0.044 | -0.002 | **0.046** | d |
| GT211 | 2 | 56 | 35 | 11 | 0.784 | 0.825 | -0.008 | 0.002 | -0.01 | d |
| GT271 | 5 | 64 | 35 | 11 | 0.600 | 0.71 | 0.097 | 0.001 | **0.096** | d |
| GT310 | 1 | 54 | 32 | 11 | 0.732 | 0.734 | 0.009 | -0.001 | 0.011 | d |
| GT575 | 2 | 56 | 35 | 8 | 0.731 | 0.727 | -0.012 | 0.000 | -0.012 | d |
| TAA023 | 4 | 54 | 32 | 6 | 0.634 | 0.708 | 0.047 | 0.001 | **0.045** | b |
| Overall |  |  |  | 11.5 | 0.768 | 0.799 | 0.018 | -0.001 | **0.019** |  |

Mplex=multiplex reaction number; T_A_=annealing temperature; Cyc=number of PCR cycles; *H*_O_=observed heterozygosity; *H*_E_=expected heterozygosity; *F*_IT_= estimate of genetic differentiation between individuals in the total population; *F*_ST_= estimate of genetic differentiation between subpopulations in the total population; *F*_IS_=estimate of genetic differentiation between individuals within subpopulations. *F*_IT_, *F*_ST_, and *F*_IS_ were estimated according to Weir and Cockerham’s (1984). *F*_IS_ estimates in bold are significantly different from Hardy-Weinberg expectations. Ref = reference. a: Valsecchi and Amos (1996); b: Palsbøll *et al.* (1997); c: Bérubé *et al.* (1998); d: Bérubé *et al.* (2000)

**Table S3** Gene and Protein IDs for selected reference species

|  | **MDH-1** |  | **MPI** |  |
| --- | --- | --- | --- | --- |
| Species | Gene ID | Protein ID | Gene ID | Protein ID |
| Human (*Homo sapiens*) | 154200 | P40925 | 4351 | P34949 |
| Rat (*Rattus norvegicus*) | 24551 | O88989 | 300741 | Q68FX1 |
| Dog (*Canis lupus familiaris*) | 474614 | E2QV08 | 478369 | F1P6B2 |
| Pig (*Sus scrofa*) | 396894 | P11708 | 100154551 | F1SJ30 |
| Cow (*Bos Taurus*) | 535182 | Q3T145 | 280865 | NP_001030361.1 |

Gene ID was obtained from the NCBI Gene database ([www.ncbi.nlm.nih.gov](http://www.ncbi.nlm.nih.gov)). Protein ID was obtained from the UniProtKB database ([www.uniprot.org](http://www.uniprot.org)). Protein ID for cow was obtained from the NCBI Protein database ([www.ncbi.nlm.nih.gov](http://www.ncbi.nlm.nih.gov))

.

**Table S4** Characteristics of the primer pairs designed to amplify and sequence the exons and partial introns of the *MDH-1* and *MPI* nuclear genes of the North Atlantic fin whale.

| **PCR**  **Locus** | **Forward primer** | **F primer (5'-3')** | **Reverse primer** | **R primer (5'-3')** | **Prg.** | **T_A_** | **Tm_E_** | **Cyc** | **bp** |
| --- | --- | --- | --- | --- | --- | --- | --- | --- | --- |
| MPI 1 | MPI12F | ACTACATTTCCCATGAAGGTC | Bp.MPI.SeqA.00527R | AGTGGGAATACTGGGGATAC | 1 | 56 | 45 | 35 | 650 |
| MPI 2 | Bp.MPI.SeqA.00214F | GCGATGGGAATTGAGCCGTTAT | Bp.MPI.SeqA.00711R | CCTTGAGTGGAGGAGTACCTTG | 1 | 56 | 45 | 35 | 550 |
| MPI 3 | Bp.MPI.intron2.00855F | ATTGGGAGTTGACAGGCTT | Bp.MPI.intron3.00107R | GGTCTTGGGAGTCCCATTT | 1 | 56 | 45 | 35 | 450 |
| MPI 4 | Bp.MPI.SeqC.00022F | AGAGCAAGGTCTGGGTGAGATT | BP.MPI.SeqC.00499R | AAGATGGAACCGGCAAAAGTGG | 1 | 56 | 45 | 35 | 500 |
| MPI 5 | Bp.MPI.SeqC.00362F | GGGCTGGTTGACCTCATGAATTCT | BP.MPI.SeqC.00796R | GAACCCTATCCCCACAGCCTAGT | 1 | 56 | 45 | 35 | 500 |
| MPI 6 | MPI6F | GCTGGAATATTAAGGAAGGCT | MPI6R | GAACCTGGGAGGTCAGAG | 7 | 56 | 45 | 32 | 500 |
| MPI 7 | MPI78F | GCTCATGGCACTCAAGTTA | Bp.MPI.SeqE.0538R | GGGCAGAAAGAAACAGCAAAG | 1 | 56 | 45 | 35 | 650 |
| MPI 8 | Bp.MPI.SeqE.00242F | TGCCAGACTTCACCGTTATG | MPI78R | CAGCTCTTCCAGGGTGTA | 1 | 56 | 45 | 35 | 700 |
| MPI 78 | MPI78F | GCTCATGGCACTCAAGTTA | MPI78R | CAGCTCTTCCAGGGTGTA | 12 | 54 | 75 | 35 | 900 |
| MDH1 1 | Hs.MDH1.exon1.-00080F | GCAGCGTAAACTACAGTTCCCA | Hs.MDH1.intron1.00115R | GAAGATGAGTCCGAGCTTGCCAAA | 1 | 56 | 45 | 35 | 300 |
| MDH1 2 | Tt.MDH1.exon1.-00127F | TTCTACTAAGCCAGATGCGA | Tt.MDH1.intron1.00282R | GCTGATTCAGCAACTTTCTC | 1 | 56 | 45 | 35 | 500 |
| MDH1 3 | Bp.MDH1.intron2.00678F | GCCTGATGTTAGACACCTGA | Tt.MDH1.intron2.00074R | CTAACTGAGAGGCCAGCTAA | 8 | 60 | 45 | 35 | 350 |
| MDH1 4 | Bt.MDH1.intron2.02752F | GGAAGAGTTACTGGGTAGATTCA | Bp.MDH1.intron4.00055R | GCAGAAGTCTCAAATGCTGAT | 1 | 56 | 45 | 35 | 300 |
| MDH1 5 | Tt.MDH1.intron3.01704F | TAGAACGGAGGATAGAAACC | Tt.MDH1.intron4.00228R | CCACTGTACTAAGAGGACATT | 7 | 56 | 45 | 32 | 550 |
| MDH1 6 | Bp.MDH1.intron5.007817F | CCTGAAGTGCTTTCTGAACCT | Bp.MDH1.intron6f.00119R | CTGCTGTCCTTGAGTTTCCT | 7 | 56 | 45 | 32 | 900 |
| MDH1 7 | Bp.MDH1.intron6f.00321F | CAGGAAGACTGGTGCTTCTAA | Bp.MDH1.intron7r.01040R | CATTCATCTGTCGATGGACACT | 9 | 56 | 75 | 29 | >1000 |
| MDH1 8 | Bp.MDH1.intron7r.01716F | TGGAAGTGCCTGATAATGGT | Bp.MDH1.intron8.00152R | CCTGAAGTTGTGCTTCACAT | 3 | 56 | 75 | 35 | 400 |
| MDH1 9 | Bp.MDH1.intron8.00491F | CTAAGTTGTTGCTTGCTGGAA | Tt.MDH1.exon8.00259R | CAAATCTGTACCAGGAAGCAC | 16 | 58 | 45 | 32 | 550 |

T_A_=Annealing temperature; Tm_E_: Extension time; Cyc: the number of PCR cycles; bp: length of the PCR product in base pairs.

**Table S5** Characteristics of putative alternative splice isoforms of fin whale *MDH-1* and *MPI*. In the three *MDH-1* isoforms I-III, isoform I is the canonical isoform and isoform III appears to be specific to humans as exon 1 is not conserved in other mammals (data not shown). The human *MPI* protein exists in four putative isoforms I-IV of which isoform I is the canonical isoform. Length, isolelectric point (pI), molecular weight (Mw), net change, and instability index was estimated in ProtParam by assuming homology between known human isoforms and the deduced fin whale amino acid sequences. Verification is the level of isoform verification in humans

| **Isoform** | **Length** | **pI** | **Mw** | **Charge** | **Instability** | **Verification** | **UniProtID** |
| --- | --- | --- | --- | --- | --- | --- | --- |
| *MDH-1* I | 334 | 6.16 | 36450.2 | -2 | 20.19 | Transcript | P40925 |
| *MDH-1* II | 245 | 8.88 | 27021.0 | 5 | 17.57 | Transcript | P40925-2 |
| *MDH-1* III | 352 | - | 38628.0 | - | - | Transcript | P40925-3 |
| *MPI* I | 423 | 5.57 | 46748.1 | -8 | 46.17 | Protein | P34949-1 |
| *MPI* II | 362 | 5.06 | 39893.1 | -12 | 43.86 | Not verified | P34949-2 |
| *MPI* III | 373 | 5.66 | 41222.8 | -7 | 44.64 | Transcript | B4DW50 |
| \| *MPI* IV \| \| --- \| | 267 | 6.31 | 29725.2 | -3 | 41.54 | Transcript | Q8NHZ6 |

**Table S6** Protein residues known or predicted to be targets of post-translational modifications (PTMs).

| **PTM type** | **Residue** | **Peptide** | **Species** | | **Method** | **Reference** |
| --- | --- | --- | --- | --- | --- | --- |
| **MDH-1** |  |  |  | |  |  |
| Acetylation | 2 | S | Hs | | MS | UniProtKB |
|  | 103 | K | Hs | | MS | PhosphoSite, UniProtKB, PHOSIDA |
|  | 118 | K | Hs | | MS | PhosphoSite, UniProtKB, PHOSIDA |
|  | 121 | K | Hs | | MS | PHOSIDA |
|  | 298 | K | Hs | | MS | PhosphoSite, UniProtKB, PHOSIDA |
| Phosphorylation | 119 | Y | Hs, Mm, Rn | | MS | PhosphoSite |
|  | 123 | S | Bp | | P | NetPhos |
|  | 143 | S | Hs | | ? | PHOSIDA |
|  | 146 | S | Hs | | ? | PHOSIDA |
|  | 188 | S | Mm | | SS | Huttlin *et al.* 2010 |
|  | 192 | Y | Bp | | P | NetPhos |
|  | 210 | Y | Hs, Mm | | MS | PhosphoSite, UniProtKB |
|  | 217^1^ | S | Mm, Bp | | SS | Huttlin *et al*. 2010, PhosphoSite, NetPhos |
|  | 241 | S | Hs, Mm, Rn | | SS | Huttlin *et al.* 2010, PhosphoSite, NetPhos, UniProtKB |
|  | 242 | S | Hs, Mm | | MS | PhosphoSite, PHOSIDA |
|  | 245 | S | Hs | | ? | PHOSIDA |
|  | 309^1^ | S | Hs, Mm | | SS | Huttlin *et al.* 2010, PhosphoSite |
|  | 316 | T | Bp | | P | NetPhos |
|  | 331 | S | Hs | | ? | PHOSIDA |
|  | 332^2^ | S | Hs | | ? | PHOSIDA |
|  | 333 | S | Hs | | MS | PhosphoSite |
| Sumoylation | 220 | K | Bp | | P | SUMOsp |
|  |  |  |  | |  |  |
| **MPI** |  |  |  | |  |  |
| Acetylation | 2 | A | Hs | | MS | UniProtKB |
| Phosphorylation | 182^3^ | S | Bp | | P | NetPhos |
|  | 187 | S | Bp | | P | NetPhos |
|  | 329 | S | Bp | | P | NetPhos |
|  | 334 | Y | Bp | | P | NetPhos |
|  | 336^4^ | S | Bp | | P | NetPhos |
|  | 355 | S | Bp | | P | NetPhos |
|  | 359^5^ | Y | Mm | | MS | PhosphoSite |
|  | 389^6^ | S | Bp | | P | NetPhos |
|  | 405^7^ | S | Bp | | P | NetPhos |
| Sumoylation | 203^8^ | K | Bp | | P | SUMOsp |
|  | 349 | K | Bp | | P | SUMOsp |
|  | | | |  |  |  |

Bp = fin whale; Hs = human; Mm = mouse; Rn = rat; 1= not detected in liver; 2 = A residue in fin whale, bottlenose dolphin and cow (Figure S1); 3 = T residue in human; 4= Y residue in dog and V residue in pig; 5 = only detected in skin cancer cells; 6 = S residue in fin whale, humpback whale and dolphin, but P residue in human, rat, dog, pig and cow; 7 = L residue in rat; 8 = N residue in pig; SS = detected using site specific methods; MS = detected using mass spectrometry; P = predicted from primary protein sequence

**Table S7** *MDH-1* and *MPI* enzyme loci polymorphisms reported for other fin whale populations and cetacean species.

|  |  |  | *MDH-1* |  |  | *MPI* |  |  |  |
| --- | --- | --- | --- | --- | --- | --- | --- | --- | --- |
| Baleen whales | Locality | Tissue | n | alleles | MAF | n | alleles | MAF | Ref |
| Bryde's whale | Solomon Is. | L | 8 | 1 |  | 8 | 1 |  | a |
|  | Fiji | L | 158 | 1 |  | 243 | 1 |  | a |
|  | Indonesia | L | 118 | 1 |  | 118 | 1 |  | a |
|  | Madagascar | L | 105 | 1 |  | 105 | 1 |  | a |
|  | North Pacific | L | 1333 | 2 | 0.020 | 175 | 1 |  | a |
|  | Peru coast | L | 125 | 2 | 0.024 | 120 | 1 |  | a |
| Fin whale | Iceland | L | 283 | 2 | 0.290 | 268 | 3 | 0.106; 0.009 | b |
|  | Spain west | L | 46 | 2 | 0.674* | 40 | 2 | 0.562* | b |
|  | Canada east | S | 24 | 2 | 0.687* | 24 | - | - | c |
|  | Iceland | S | 24 | 2 | 0.042 | 24 | 1 |  | c |
|  | Norway | S | 17 | 2 | 0.118 | 19 | - | - | c |
|  | Antarctic | L | 61 | 1 |  |  |  |  | a |
|  | North Pacific | L | 50 | 1 |  |  |  |  | a |
|  | Spain west | L | 89 | 1 |  | 89 | 1 |  | a |
| Minke whale (common) | Greenland west | L | 80 | 1 |  |  |  |  | d |
|  | Japan coast | L | 34 | 1 |  |  |  |  | e |
|  | Brazil coast | L | 195 | 2 | 0.003 |  |  |  | a |
|  | Japan coast | L | 404 | 1 |  | 385 | 2 | 0.008 | a |
|  | Korea coast | L | 46 | 2 | 0.022 | 46 | 1 |  | a |
| Minke whale (southern) | Antarctica | L | 8979 | 2# | 0.007 | 1324 | 3 | 0.033; 0.009 | a |
| Sei whale | Iceland | L | 101 | 2 | 0.087 | 100 | 3 | 0.042; 0.031 | b |
|  | Antarctic | L | 948 | 2 | 0.002 | 254 | 1 |  | a |
|  | North Pacific | L | 48 | 1 |  |  |  |  | a |

**Table S7** continued

|  |  |  | *MDH-1* |  |  | *MPI* |  |  |  |
| --- | --- | --- | --- | --- | --- | --- | --- | --- | --- |
| Toothed whales | Locality | Tissue | n | alleles | MAF | n | alleles | MAF | Ref |
| Bottlenose dolphin | Japan coast | L | 35 | 1 |  | 35 | 2 | 0.275¤ | f |
| Dall's porpoise | Japan coast | L | 54 | 2 | 0.013 | 54 | 4 | 0.175; 0.025; 0.025 | f |
| Dall's porpoise | North Pacific | L | 483 | 2 | 0.013 | 483 | 2 | 0.275 | f |
| False killer whale | Japan coast | L | 31 | 1 |  | 31 | 1 |  | f |
| Finless porpoise | Japan coast | L | 3 | 1 |  | 3 | 1 |  | f |
| Giant beaked whale | Japan coast | L | 9 | 1 |  | 9 | 2 | 0.170 | f |
| Harbour porpoise | Japan coast | L | 3 | 1 |  | 3 | 2 | 0.170 | f |
| Long-finned pilot whale | Faroe Is. | M | 633 | 1 |  | 633 | 2 | 0.451 | g |
| Melon-headed whale | Japan coast | L | 6 | 1 |  | 6 | 1 |  | f |
| Pacific white-sided dolphin | Japan coast | L | 30 | 1 |  | 30 | 2 | 0.475¤ | f |
| Pantropical spotted dolphin | Japan coast | L | 370 | 1 |  | 370 | 1 |  | f |
| Rough-toothed dolphin | Japan coast | L | 10 | 1 |  | 10 | 1 |  | f |
| Short-finned pilot whale | Japan coast | L | 39 | 2 | 0.010 | 39 | 1 |  | f |
|  | Japan north | L | 154 | 1 |  | 154 | 1 |  | i |
|  | Japan south | L | 167 | 2 | 0.024 | 167 | 1 |  | i |
| Striped dolphin | Japan coast | L | 183 | 1 |  | 183 | 4 | 0.050; 0.030; 0.030¤ | f |
|  | Japan coast | L | 40 | 1 |  |  |  |  | h |

MAF=minor allele frequency; Ref=reference; L=liver; M=muscle; S= skin; a=Wada and Numachi ([1991](#_ENREF_144)); b=Danielsdottir *et al*. ([1991](#_ENREF_26)); c=Danielsdottir *et al*.([1992](#_ENREF_27)); d=Simonsen *et al*. ([1982b](#_ENREF_125)); e=Wada ([1983b](#_ENREF_142)); f=Shimura and Numachi ([1987](#_ENREF_122)); g=Andersen ([1988](#_ENREF_3)); h=Wada ([1983a](#_ENREF_141)); i=Wada ([1988](#_ENREF_143)); *Notice that here, the “minor allele” is in fact the major; #a third allele was reported at a frequency of 0.0003; ¤not the same allele that is major as in the other species.

**Supplementary figures**

**Figure S1**

The fin whale *MDH-1* protein aligned with protein sequences from other mammals. The humpback whale sequence was obtained from a single individual sampled in the Gulf of St. Lawrence, Canada, and sequenced together with the fin whale samples.

**Figure S2**

The fin whale *MPI* protein aligned with protein sequences from other mammals. The gap in the dolphin protein sequence is due to missing data.

**Figure S3**

Pictures of the original gel electrophorese of the two enzyme loci, *MDH-1* (a) and *MPI* (b) loci in North Atlantic fin whales. Modified from Danielsdottir ([1994](#_ENREF_25)). F = fast; S = slow; H = heterozygote.

**Figure S1**

**
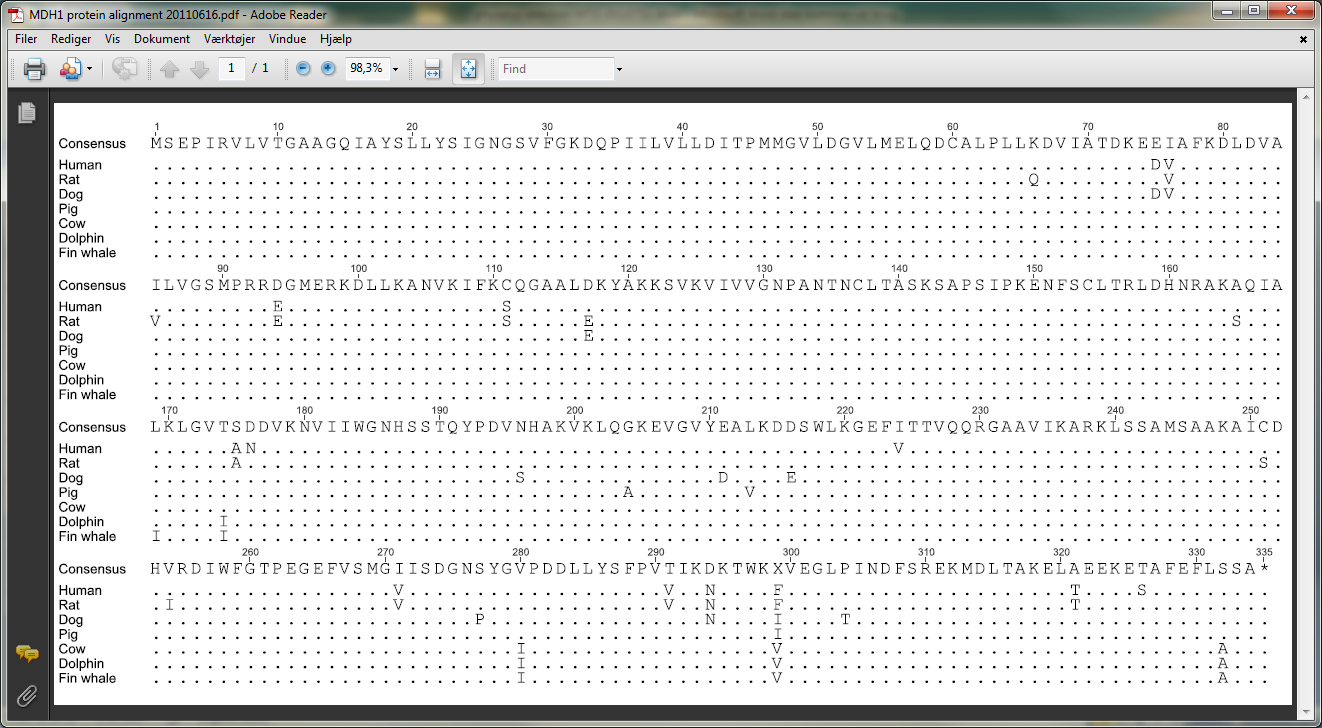
**

**Figure S2**

**
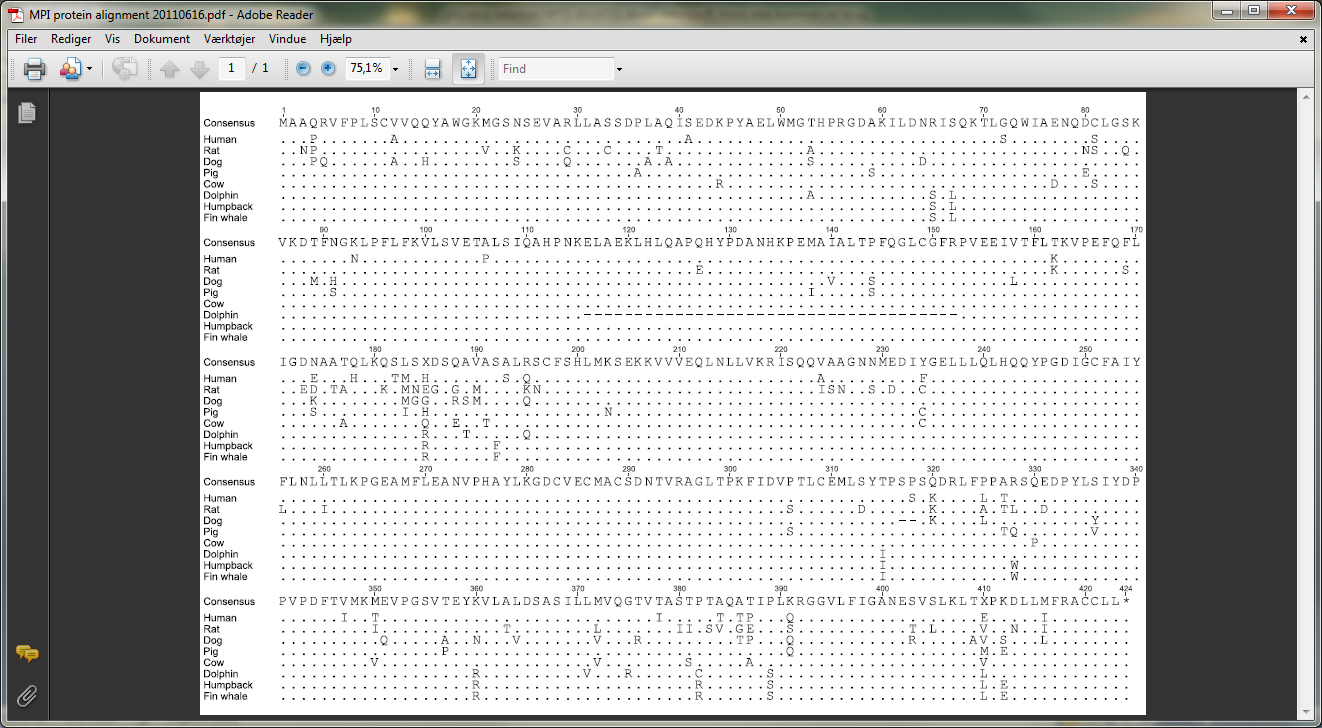
**

**Figure S3**

**a**

**
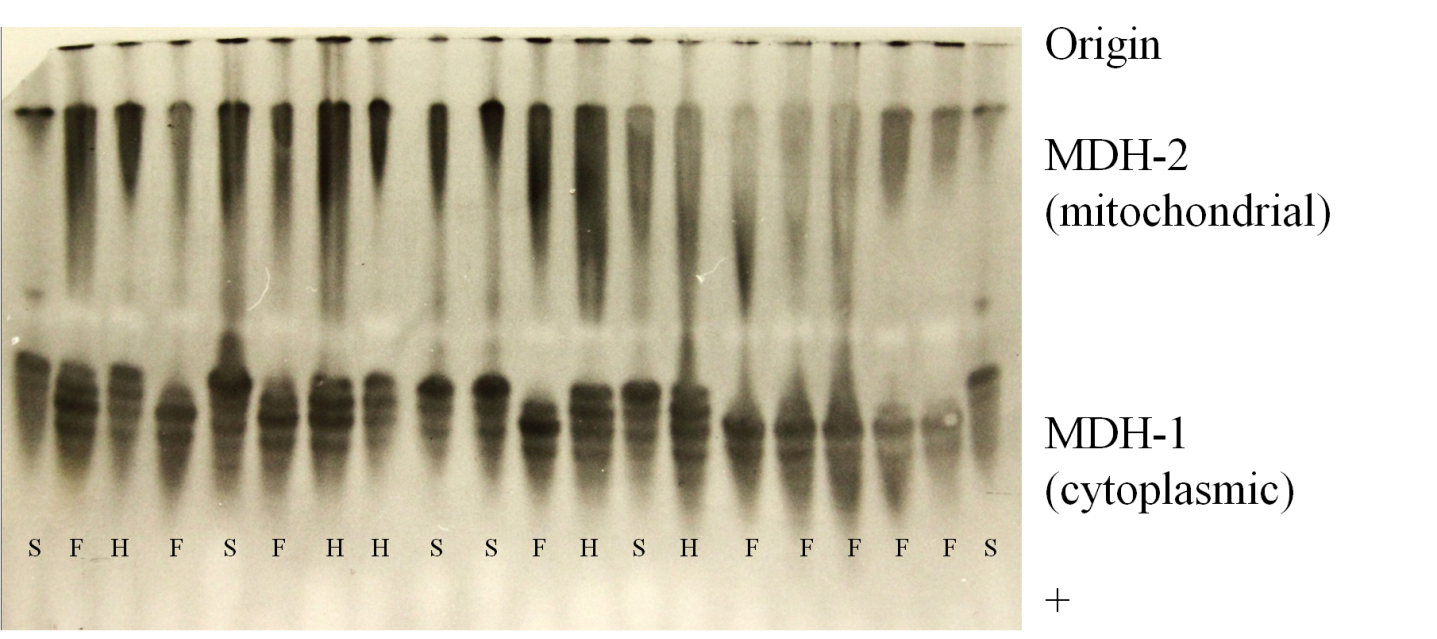
**

**b**

**
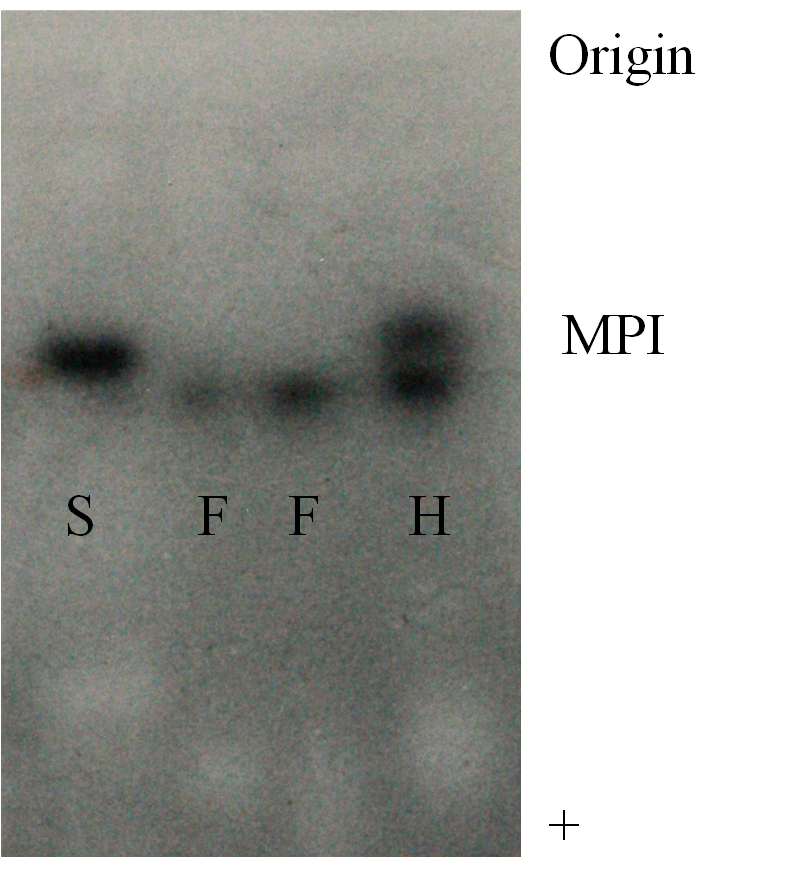
**
